# Active Fraction from Embryo Fish Extracts Induces Reversion of the Malignant Invasive Phenotype in Breast Cancer through Down-Regulation of TCTP and Modulation of E-cadherin/β-catenin Pathway

**DOI:** 10.3390/ijms20092151

**Published:** 2019-04-30

**Authors:** Sara Proietti, Alessandra Cucina, Andrea Pensotti, Pier Mario Biava, Mirko Minini, Noemi Monti, Angela Catizone, Giulia Ricci, Erica Leonetti, Abdel Halim Harrath, Saleh H. Alwasel, Mariano Bizzarri

**Affiliations:** 1 Department of Surgery “Pietro Valdoni”, Sapienza University of Rome, 00161 Rome, Italy; sara.proietti@uniroma1.it (S.P.); alessandra.cucina@uniroma1.it (A.C.); mirko.minini@uniroma1.it (M.M.); monti_noemi@libero.it (N.M.); 2 Azienda Policlinico Umberto I, 00161 Rome, Italy; 3 FAST, University Campus Bio-Medico, 00128 Rome, Italy; andreapensotti@gmail.com; 4 Scientific Institute of Research and Health Care (IRCCS) Multimedica, 20099 Milano, Italy; piermario.biava@gmail.com; 5 Department of Experimental Medicine, Sapienza University of Rome, Systems Biology Group Lab, 00161 Rome, Italy; 6 Department of Anatomy, Histology, Forensic-Medicine and Orthopedics, “Sapienza” University of Rome, 00161 Rome, Italy; angela.catizone@uniroma1.it (A.C.); erica.leonetti@uniroma1.it (E.L.); 7 Department. of Experimental Medicine, Università degli Studi della Campania “Luigi Vanvitelli”, 80138 Naples, Italy; giulia.ricci@uniroma1.it; 8 Department of Zoology, College of Science, King Saud University, Riyadh 2455, Saudi Arabia; halim.harrath@gmail.com (A.H.H.); salwasel@ksu.edu.sa (S.H.A.)

**Keywords:** tumor reversion, TCTP, embryo fish extract, cytoskeleton, E-cadherin/β-catenin, p53

## Abstract

Some yet unidentified factors released by both oocyte and embryonic microenvironments demonstrated to be non-permissive for tumor development and display the remarkable ability to foster cell/tissue reprogramming, thus ultimately reversing the malignant phenotype. In the present study we observed how molecular factors extracted from Zebrafish embryos during specific developmental phases (20 somites) significantly antagonize proliferation of breast cancer cells, while reversing a number of prominent aspects of malignancy. Embryo extracts reduce cell proliferation, enhance apoptosis, and dramatically inhibit both invasiveness and migrating capabilities of cancer cells. Counteracting the invasive phenotype is a relevant issue in controlling tumor spreading and metastasis. Moreover, such effect is not limited to cancerous cells as embryo extracts were also effective in inhibiting migration and invasiveness displayed by normal breast cells undergoing epithelial–mesenchymal transition upon TGF-β1 stimulation. The reversion program involves the modulation of E-cadherin/β-catenin pathway, cytoskeleton remodeling with dramatic reduction in vinculin, as well as downregulation of TCTP and the concomitant increase in p53 levels. Our findings highlight that—contrary to the prevailing current “dogma”, which posits that neoplastic cells are irreversibly “committed”—the malignant phenotype can ultimately be “reversed”, at least partially, in response to environmental morphogenetic influences.

## 1. Introduction

Some yet unidentified factors released by both oocyte and embryonic microenvironments demonstrated to be non-permissive for tumor development and display the remarkable ability to foster cell/tissue reprogramming, thus ultimately reversing the malignant phenotype [1,2].

Pioneer studies from the sixties showed that carcinoma cells are “reprogrammed” when injected into a mouse blastocyst, ultimately resulting in normal tissue originating from cancer cells [3]. Since then, several cancer types have been shown to undergo partial or complete reversion when exposed to embryonic environments or treated with soluble factors extracted from oocytes or embryonic cells (reviewed in [4]). Microenvironments derived from mouse, human embryonic stem cells, zebrafish (*Danio rerio*), chick, and amphibian embryo/eggs extracts were used to give an insight into the molecular reprogramming of cancer cells in response to the embryonic environment [5].

This process may entail proliferation and apoptosis rate [6], as well as partial or complete reversion of the malignant phenotype, including DNA demethylation, removal of repressive histone marks at the promoters of tumor suppressor genes, and expression of silenced genes [7,8,9,10].

These studies documented that the reversion program is a complex attempt, running through discrete steps in which differentiation that lead a stem cell toward a complete phenotypic commitment is recapitulated according to an ”inverse process” [11]. This remark substantiates the concept for which carcinoma is a caricature of the normal process of tissue renewal, or a “development gone awry” [12].

The fact that the observed reversal of malignancy in cancer cells frequently involves only some features of the malignant phenotype, evidences how limited is our knowledge of the molecular and biophysical mechanisms that orchestrated the reversion.

Namely, only few factors extracted from embryo kept during a well-defined period of development share the capability to trigger tumor reversion [13]. For instance, when embryonal carcinoma cells were injected into 8- to 15-day mouse embryos, it was found that the ability to abolish malignancy was inversely proportional to the age of the embryo at the moment of carcinoma cell transplantation [14].

Indeed, the release of morphogenetic factors significantly differs according to the different stages of embryonic development [15] and we can truthfully postulate that cancer cells can only be sensitive to only some signaling molecules depending on the differentiated stage of the tumor itself. Moreover, the participation of non-molecular factors, i.e., biophysical constraints shaping the overall architecture of cell and tumor tissue, cannot be discarded and should instead be considered mandatory to ensure a full reversion of the malignant phenotype [16], given that reprogramming can also be achieved only through manipulation of the biophysical properties of the microenvironment [17].

We have already shown that stage developmental factors extracted from embryos of Zebrafish can efficiently arrest cancer proliferation and induce apoptosis, both in vitro [18] and in vivo [19]. Moreover, sublingual administration of Zebrafish extracts showed to improve response to conventional chemotherapy while reducing the incidence of drug-resistance in a pilot study on human colon cancer patients [20].

Mature oocytes and undifferentiated embryonic stem (ES) cells contain reprogramming factors (proteins, RNAs, lipids, small molecules) that enable these cells to reprogram a somatic nucleus to pluripotency [21]. However, compelling evidence is still lacking. Nevertheless, it can be inferred from in vivo studies that embryo/oocyte factors are likely low-weight soluble components, easily absorbable through the mucosa. Co-culture of breast cancer cells with embryonic mesenchyme from early stage mammary glands decreases tumor cell proliferation while stimulating acinus differentiation, as reported by a few scientific studies. Namely, both soluble and insoluble components of embryo microenvironment have demonstrated the ability to reverse neoplastic progression and “reboot” breast cancer, resulting in at least partial normalization of tumor cells [22,23].

Over the last decades, Zebrafish has proven to be a powerful model in cancer research. Indeed, Zebrafish displays more than 80% of all human disease-related genes, indicating that many human diseases can, in fact, be modeled in Zebrafish. Namely, an impressive body of studies demonstrated that Zebrafish can serve as a useful model to investigate tumor driving as well as anti-tumor mechanisms [24]. Moreover, cancer research in Zebrafish particularly benefits from the many genetic tools and transgenic strains established by the Zebrafish community over the years. 

Herewith we seek to determine the stages of Zebrafish embryo development that display the most significant activity in inducing the reversal of prominent malignant features, like proliferation, migrating capacity, and invasiveness.

## 2. Results

### 2.1. Recognizing the Most Effective Embryo Fish Extract Fraction

We performed a preliminary screening to identify the most effective embryo fraction (EF). Biological activity was ascertained by considering as prominent parameter the reduction in cell viability, assessed with the Sulforhodamine B (SRB) colorimetric assay. Two concentrations (0.3 and 3.0 μg/mL) of each EF were tested in breast cancer cell lines—MCF7 and MDA-MB-231—at different time points (24, 48, and 72 h). This preliminary survey identified F6 (corresponding to the 20-somite stage) to be the most active fraction, and significant effects were also recorded in F4 and F5, for both cell lines. No significant differences were found between 0.3 and 3 μg/mL concentrations (Supplementary Materials Figures S1 and S2; Tables S1 and S2). Hereafter, only the F6 at 0.3 μg/mL was utilized in the succeeding experimental phases.

### 2.2. Embryo Extract Stimulates Apoptosis

**Figure 1:**
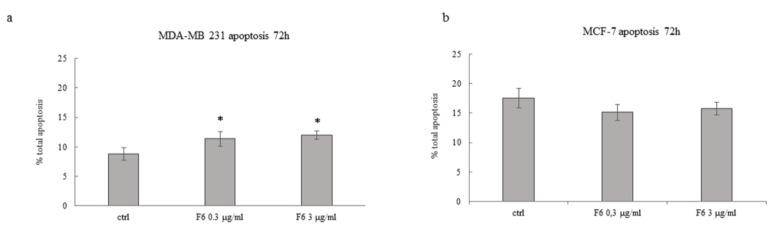
Effect of Zebrafish embryo F6 fraction on apoptosis of MDA-MB-231 (**a**) and MCF-7 (**b**) cells after 72 h of treatment with F6 at 0.3 and 3 μg/mL. Histograms showing the percentage of apoptotic cells; each column represents the mean value ± SD of four independent experiments. * *p* < 0.05 versus ctrl by ANOVA followed by Bonferroni post-test.

Besides the reduction in cell viability as assessed with the SRB test, we investigated if EFs could foster the apoptosis rate. As expected, fractions from F1 to F5 did not induce any significant increase in apoptosis either in MCF7 or MDA-MB-231 cancer cells, at any of the time points we considered (data not shown). Instead, F6 at both 0.3 and 3 μg/mL significantly raise the apoptosis rate in MDA-MB-231 cells, while in MCF7 cells only a trend toward increased apoptosis was observed, albeit not significant (Figure 1). Given that at 72 h 5FU kills almost all cells, the possible additive effect of F6 cannot be ascertained when F6 was associated with the chemotherapic drug. Overall, data obtained with both the SRB and the MUSE test demonstrated that some embryo extracts could efficiently impair cancer cell viability.

### 2.3. Embryo Extract Reduces Cancer Cell Proliferation

**Figure 2:**
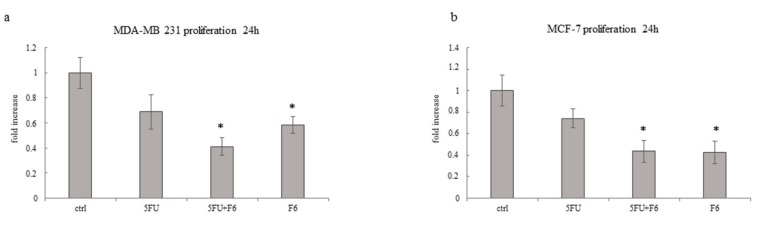
Effect of 5FU, 5FU+F6, and F6 on proliferation of MDA-MB-231 (**a**) and MCF-7 (**b**) cells. Cell proliferation was determined after 24 h of treatment by cell count assays performed by a particle count and size analyzer. Values, expressed as fold increase of control value considered as 1, are means of three independent experiments performed in triplicate, with SD represented by vertical bars. * *p* < 0.05 versus ctrl by ANOVA followed by Bonferroni post-test.

Cell proliferation was investigated in MDA-MB-231 and MCF-7 cells at 24 h by comparing data recorded in cells treated with 5FU or F6 alone and in association. As shown in Figure 2, in both cell lines 5FU slightly reduces cell proliferation. F6 and 5FU+F6 significantly decreased cell growth to less than 60% of control values. Moreover, in MDA-MB-231 cells, the association 5FU+F6 further decreased cell proliferation compared to F6 alone, even if without statistical relevance. These findings evidenced that F6 significantly slows down cancer proliferation and most likely amplifies the cytostatic effect of 5FU.

### 2.4. Embryo Extract Antagonizes Cancer Cell Invasiveness and Migrating Capability

**Figure 3:**
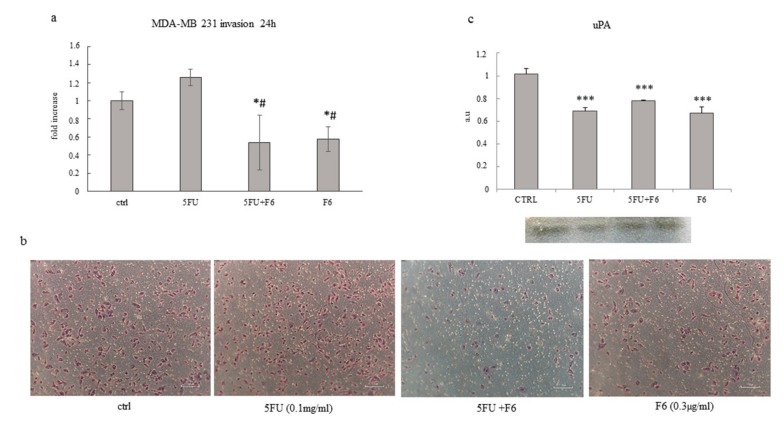
Effect of 5FU, 5FU+F6, and F6 on invasion in MDA-MB-231 cells. Transwell invasion assay (**a**,**b**) and urokinase plasminogen activator (uPA) levels (**c**) was performed in MDA-MB-231 cells untreated (ctrl) and treated with 5FU, 5FU+F6, and F6 for 24h. Values, expressed as fold increase of control value considered as 1, are means of three independent experiments performed in duplicate, with SD represented by vertical bars. * *p* < 0.05; *** *p* < 0.001 versus ctrl; ^#^ *p* < 0.05 versus 5FU by ANOVA followed by Bonferroni post-test. Images were obtained by optical microscopy, with 100× magnification.

**Figure 4:**
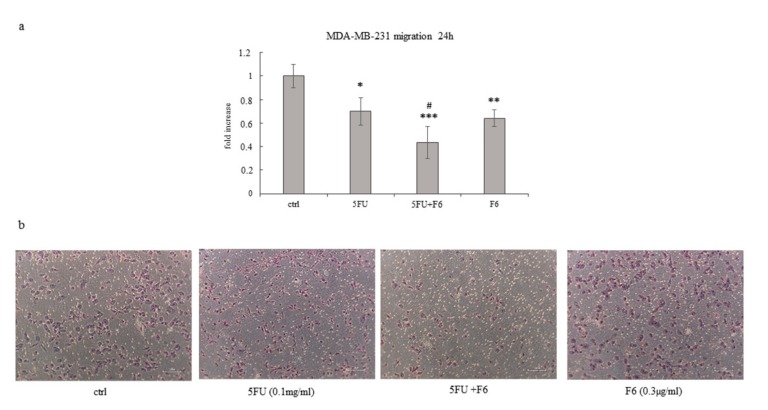
Effect of 5FU, 5FU+F6, and F6 on migration in MDA-MB-231 cells. Transwell migration assay (**a**,**b**) was performed in MDA-MB-231 cells untreated (ctrl) and treated with 5FU, 5FU+F6, and F6 for 24 h. Values, expressed as fold increase of control value considered as 1, are means of three independent experiments performed in duplicate, with SD represented by vertical bars. * *p* < 0.05; ** *p* < 0.01; *** *p* < 0.001 versus ctrl; ^#^ *p* < 0.05 versus 5FU by ANOVA followed by Bonferroni post-test. Images were obtained by optical microscopy, with 100× magnification.

**Figure 5:**
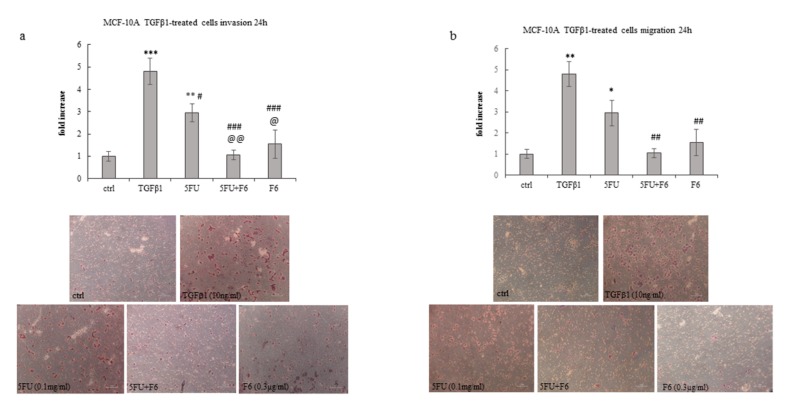
Effect of 5FU, 5FU+F6, and F6 on invasion (**a**) and migration (**b**) in MCF-10A cells. Transwell assays were performed in MCF-10A cells untreated (ctrl), and pre-treated with TGF-β1 for 5 days. TGF-β1 stimulated MCF-10A cells were then treated with 5FU, 5FU+F6, and F6 for 24 h. Values, expressed as fold increase of control value considered as 1, are means of three independent experiments performed in duplicate, with SD represented by vertical bars. * *p* < 0.05; ** *p* < 0.01; *** *p* < 0.001 versus ctrl; ^#^ *p* < 0.05; ^##^ *p* < 0.01; ^###^ *p* < 0.001 versus TGF-β1; ^@^ *p* < 0.05; ^@@^ *p* < 0.01 versus 5FU by ANOVA followed by Bonferroni post-test. Images were obtained by optical microscopy, with 100× magnification.

To ascertain to what extent F6 can significantly reverse the malignant phenotype, we plan to investigate some remarkable parameters belonging to the macroscopic–mesoscopic level, where microscopic elements are “channeled” and organized in a coherent manner in producing macroscopic features, as recorded by macroscopic parameters [25]. Indeed, the mesoscopic approach strives to “capture” the self-organizing process, which in turn will lead to the emergence of specific system’s properties [26]. Therefore, we evaluate invasiveness and migrating capability in the highly malignant cell line MDA-MB-231, given that MCF7 cells display only minimal invasive capacity. We observed that F6 dramatically reduced invasiveness below to 60% as recorded in untreated cells, while 5FU had no effect (Figure 3a,b). Both MMP2 and MMP9 have been measured to investigate their potential involvement in the observed inhibition of invasiveness. As a result, MMP9 was reduced in both F6 and 5FU+F6, while MMP2 shows a slight increase in both conditions (data not shown). Overall, such changes were of little significance and we decided to look at uPA to ascertain if invasiveness reduction in treated samples could be attributed to uPA modulation. Indeed, inhibition of invasive phenotype was further confirmed when urokinase plasminogen activator (uPA) levels were investigated in conditioned media of MDA-MB-231 cells. During tumor progression, uPA, after binding to its receptor (uPAR), activates a cascade of proteases, ultimately leading to the degradation of the basement membrane, thus fostering tumor cell invasiveness. Decreasing of uPA in breast cancer cells dramatically reduces the wound healing, migratory, invasive, and adhesive capacity of cancer cells [27]. In our experiments, uPA levels were significantly reduced after 24 h in 5FU- and F6-treated cells, while no additive effects were observed with the association of both (Figure 3c).However, inhibition of invasiveness in MDA-MB-231 cells can only partially be explained by downregulation of a single molecular factor, alike uPA. Indeed, in 5FU-treated cells, despite uPA reduction, invasiveness remains unchanged. Probably other factors, including cytoskeleton modifications (i.e., those involving migratory/invasive structures, like pseudopodia) play a major role. Furthermore, migration was highly hindered in both 5FU- and F6-treated groups (Figure 4a,b). Remarkably, F6 was even more efficient than 5FU in inhibiting migratory capability, while the association of both F6+5FU were shown to exert additive effects. Embryo factor demonstrated thus to be even more effective than conventional chemotherapy in reversing prominent malignant features like invasiveness and migratory behavior. To ascertain if this effect could be traced back to the epithelial–mesenchymal (EMT) features harbored by invasive cancer cells, we investigated the F6 inhibitory effects on a previously studied model of normal breast cells (MCF10A), which had been committed to EMT upon TGF-β stimulation [28]. Briefly, the immortalized, not transformed MCF10A breast cell line was treated with TGF-β1 for five days. Both invasiveness and motility of MCF10A cells increased to fivefold under these conditions (Figure 5a,b). Addition of 5FU only partially mitigated that increase, while F6 almost completely nullified the TGF-β increase. This finding specifically evidenced that F6 was able in interfering with the acquisition of the invasive, EMT-dependent phenotype, independently from either the malignant or the benign hallmark of cells under study.

### 2.5. Cytoskeleton Remodeling

**Figure 6:**
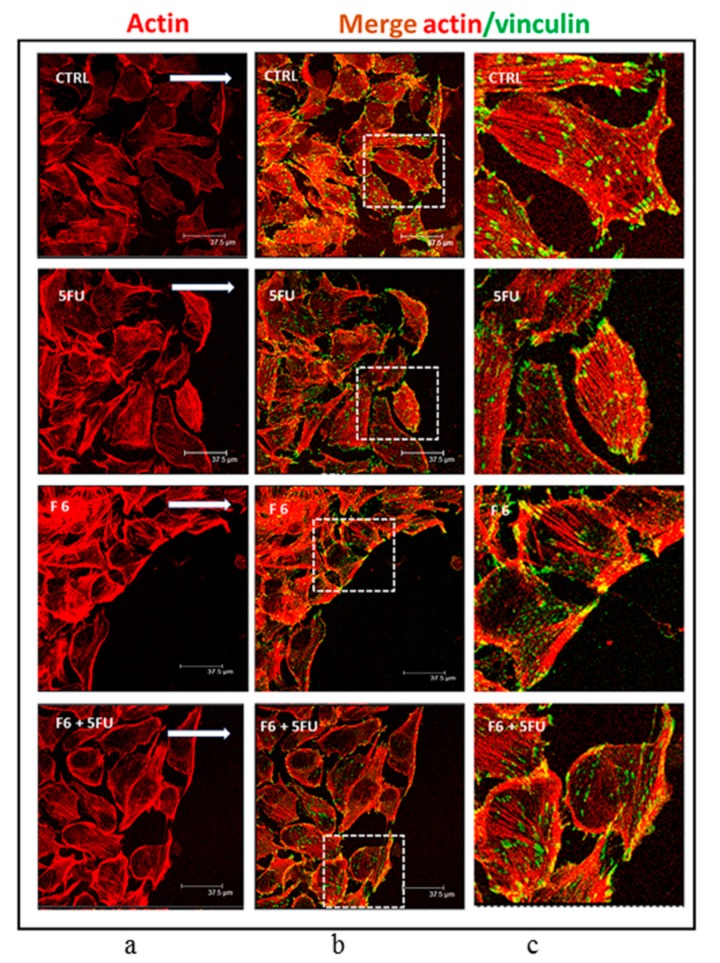
Distribution pattern of vinculin and F-actin in wound-healing assay performed on MDA cells cultured in control condition or exposed to 5FU, F6, and 5FU+F6. Confocal microscopy analysis of F-actin staining with rhodamine-phalloidin (red signal, (**a**) column) merged with anti-vinculin immunofluorescence (FITC/green signal, b column) on MDA cells subjected to wound healing assay, and cultured with or without 5FU, F6, 5FU+F6. The white arrows in the images of the left column indicate the direction of cellular movement toward the gap. In column (**c**) we reported higher magnification of the merging pictures shown in column (**b**).

**Figure 7:**
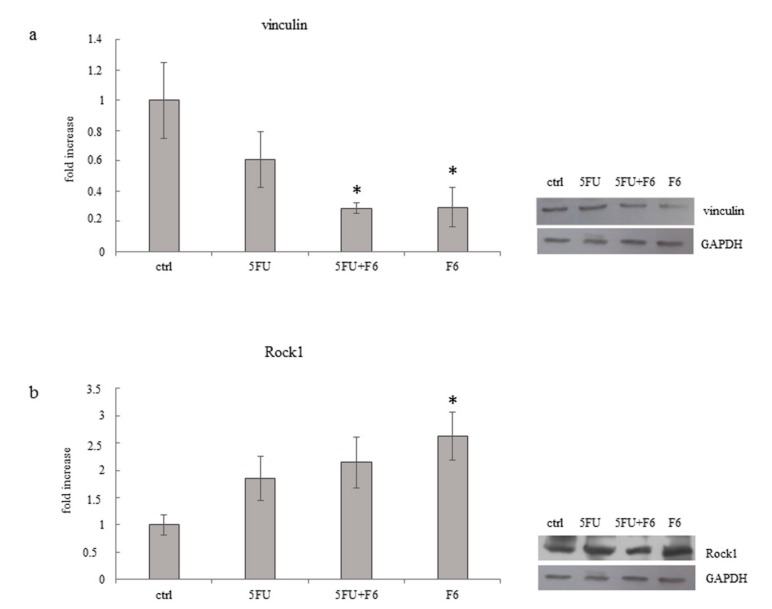
Effect of 5FU, 5FU+F6, and F6 on expression of vinculin (**a**) and Rock1 (**b**) in MDA-MB-231 cells. Columns represent densitometric quantification of optical density (OD) of specific protein signal normalized with the OD values of GAPDH served as a loading control and they are expressed as fold increase of control value considered as 1. Each column represents the mean value ± SD of three independent experiments. * *p* < 0.05 versus ctrl by ANOVA followed by Bonferroni post-test. Representative western blot analysis relating to vinculin and Rock1expression in MDA-MB-231 cells untreated (ctrl) and treated with 5FU, 5FU+F6, and F6 for 24 h. GAPDH was used as loading control.

Changes in the migratory/invasive phenotype are indeed mirrored by cytoskeleton rearrangement under the influence of F6. While both control and 5FU-treated MDA-MB-231 cells harbored a dense texture of stress fibers, with actin filaments distributed all along the cytosol, in F6-treated cells actin was predominantly concentrated along the membrane border (Figure 6a). Moreover, F6-treated MDA-MB-231 cells almost completely lost pseudopodia and lamellipodia, two prominent structures required by migrating/invasive cells, as evidenced in control and 5FU-treated MDA-MB-231 cells, in which polarized lamellipodia with treadmilling filaments, as well as filopodia were clearly observable. Overall, those changes enabled F6-treated cells to recover a rounded shape, with reduced spreading and smaller nucleus (Figure S3). The loss of the migratory/invasive phenotype is further confirmed when looking at the distribution of vinculin fibers and their association with actin (Figure 6b,c). Vinculin-expressing cells are able to migrate into dense three-dimensional collagen matrices that were impenetrable for vinculin knockout cells. Indeed, vinculin facilitates three-dimensional matrix invasion through up-regulation or enhanced transmission of traction forces that are needed to overcome the steric hindrance of extra-cellular matrix [29]. We observed that in F6-treated cells vinculin levels are significantly reduced (Figure 7a), namely at the membrane border, where vinculin seems to be dissociated from actin filaments. Indeed, vinculin preferentially localizes inside the cytosol, thus losing contact with actin filament and impairing the migrating and invasive capabilities of cancer cells, as previously reported [30]. This finding should be put in correlation with ROCK1 activity. In F6-treated cells, we observed a paradoxical increase in ROCK1 levels that apparently can hardly accommodate the reduced invasiveness/motility of embryo-treated cells (Figure 7b). However, ROCK1 functions differ depending on the stiffness of the substrate upon which cells are cultivated. In stiffness conditions mimicking those observed in vivo, downregulation of ROCK1 promoted cell spreading and cell migration [31]; instead, high levels of activated Rho kinase and ROCK1 are required for inhibiting motility and stabilizing E-cadherin adhesions through F-actin fibers [32]. Namely, ROCK1 activation is considered mandatory for proper reprogramming of cells [33] and for the acquisition of the correct morphological pattern in developing embryos [34]. On the contrary, in teratocarcinoma cells, the ROCK inhibitor Y-27632 promotes migration, accompanied by an apparent increase in focal complexes and lamellipodia and a decrease in focal adhesions and stress fibers. In this condition, reduced levels of vinculin amplify the motility inhibition triggered by ROCK1 increase [35]. Given that in our model we observed that ROCK1 increases and vinculin decrease, it can be hypothesized that both conditions may enhance inhibition of the migrating/invasive phenotype of MDA-MB-231 cells.

### 2.6. Embryo Extracts Promotes E-cadherin/β-catenin Redistribution behind Cell Membrane

**Figure 8:**
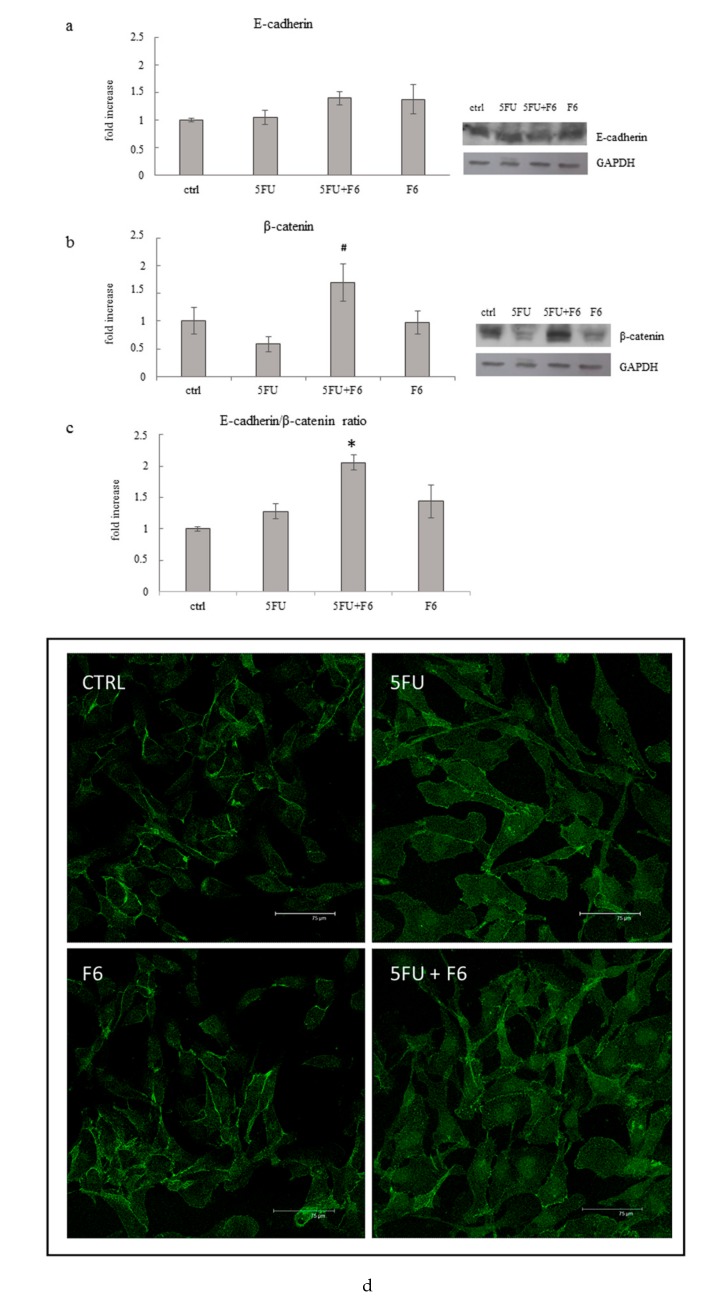
Effect of 5FU, 5FU+F6, and F6 on expression of E-cadherin (**a**), β-catenin (**b**), and E-cadherin/ β-catenin ratio (**c**) in MDA-MB-231 cells. Columns represent densitometric quantification of optical density (OD) of specific protein signal normalized with the OD values of GAPDH served as a loading control and they are expressed as fold increase of control value considered as 1. Each column represents the mean value ± SD of three independent experiments. * *p* < 0.05 versus ctrl; # *p* < 0.05 versus 5FU by ANOVA followed by Bonferroni post-test. Representative western blot analysis relating to E-cadherin and β-catenin expression in MDA-MB-231 cells untreated (ctrl) and treated with 5FU, 5FU+F6, and F6 for 24 h. GAPDH was used as loading control. (**d**) Confocal microscopy analysis of beta-catenin immunostaining (FITC/green signal) on MDA cells cultured in control condition, or with the following treatments: 5FU, F6, 5FU+F6. Distribution of β-catenin increase behind the cell membrane mostly in F6-treated cells.

MDA-MB-231 breast cancer cells showed prominent mesenchymal features, as down-regulation of E-cadherin, while β-catenin was redistributed in the cytosol and the nucleus [36]. Control cancer cells displayed low E-cadherin levels, while both 5FU- and F6-treated cells showed a trend, albeit not significant, toward increased release (Figure 8a); β-catenin increases significantly only in 5FU+F6-treated cells, while showing a slight, albeit not significant decrease in the other treatment conditions (Figure 8b). However, the E-cadherin/β-catenin ratio resulted significantly increased in the 5FU+F6-treated group (Figure 8c). Loss of either E-cadherin or β-catenin at the cell membrane contributes in disassembling E-cadherin/β-catenin complexes, through which cadherin sequesters β-catenin, preventing its dispersion into the cytoplasm, and its subsequent nuclear transcriptional activities [37].

In control cancer cells, overall β-catenin is lowered and mostly dispersed around in the cytoplasm, with only isolated spots at the membrane site of adhesion (Figure 8d). Instead, in F6-treated cells, immune staining unveiled a strong β-catenin signal, mostly located at the cell-to-cell junction level, thus indicating the restoring of the E-cadherin/β-catenin complexes.

### 2.7. Embryo Extracts Downregulate TCTP Expression in MDA-MB-231 Cells

**Figure 9:**
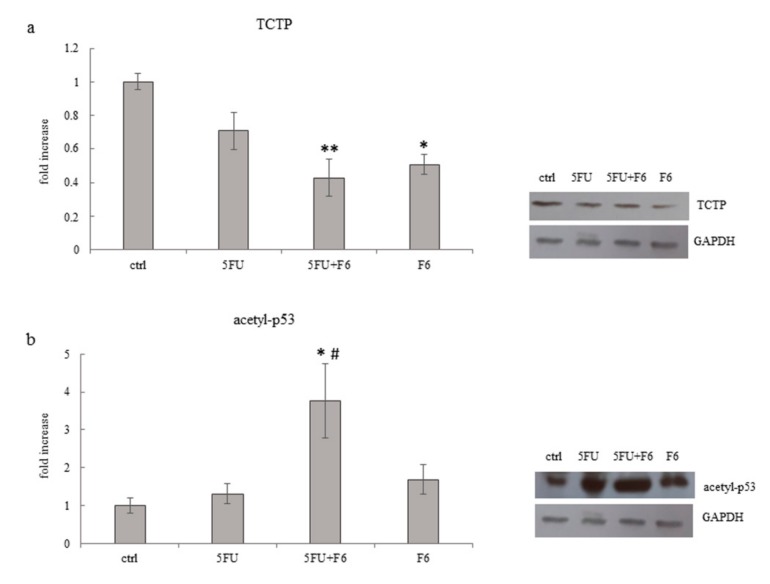
Effect of 5FU, 5FU+F6, and F6 on expression of TCTP (**a**) and acetyl-p53 (**b**) in MDA-MB-231 cells. Columns represent densitometric quantification of optical density (OD) of specific protein signal normalized with the OD values of GAPDH served as a loading control and they are expressed as fold increase of control value considered as 1. Each column represents the mean value ± SD of three independent experiments. * *p* < 0.05; ** *p* < 0.01 versus ctrl; # *p* < 0.05 versus 5FU by ANOVA followed by Bonferroni post-test. Representative western blot analysis relating to TCTP and acetyl-p53 expression in MDA-MB-231 cells untreated (ctrl) and treated with 5FU, 5FU+F6, and F6 for 24 h. GAPDH was used as loading control.

Translationally Controlled Tumor Protein (TCTP) has emerged as a critical regulator of cell fate determination, as it regulates many different biological processes, all of which may converge to a limited set of key events that control cell fate determination and namely, tumor reversion. Aberrant expression of TCTP is frequently observed in cancer cells while silencing TCTP showed to be instrumental in promoting cancer reversion in different types of cancer cells. Namely, silencing TCTP expression in breast cancer was demonstrated to restore growth and morphological patterns reminiscent of the outgrowths generated by normal mammary epithelial cells [38]. Down-regulation of TCTP is usually accompanied by an increase in p53 levels, suggesting thus that up-regulation of p53 is required for enacting the reversion process. Indeed, it has been proposed that the modulation of the TCTP-p53 axis is a pre-requisite for triggering tumor reversion [39]. In our model, F6-treated cells showed a significant down-regulation of TCTP, while in 5FU-treated cells only a slight, not significant decrease in TCTP levels was found (Figure 9a). As expected, p53 increases in treated samples. It is worth noting that the increase specifically involves the acetylated form of p53, i.e., the active form of p53, which is resilient to MDM2-dependent degradation [40] (Figure 9b).

## 3. Discussion

In the present study, we showed that unknown molecular factors extracted from Zebrafish embryos during specific developmental phases (20 somites) significantly antagonize proliferation of breast cancer cells, while reversing some prominent aspects of the malignant phenotype. Embryo extracts reduce cell proliferation, enhance apoptosis, and dramatically inhibit both invasiveness and migrating capabilities of cancer cells. Counteracting the invasive phenotype is a relevant issue in controlling tumor spreading and metastasis. Moreover, such effect is not limited to cancerous cells as embryo extracts were also effective in inhibiting migration and invasiveness displayed by normal breast cells undergoing epithelial–mesenchymal transition upon TGF-β1 stimulation. The reversion program, as previously reported by several studies, involves downregulation of TCTP and the concomitant increase in p53 levels.

TCTP is a key player in the process of tumor reversion, the process in which a tumor cell is transformed into a revertant cells by losing its malignant traits—uncontrolled growth, invasiveness, and metastasis-forming capability—through the activation of a complex cascade of biochemical events, including cytoskeleton remodeling, pathways modulation, and even gene reprogramming [38].

TCTP knockdown in primary mammary tumor cells from ErbB2 transgenic mice resulted in increased p53 expression and fewer stem cell-like cancer cells, while in breast cancer patients a high-TCTP status is associated with aggressive tumors and predicts a poor prognosis [41]. Downstream to TCTP inhibition, EMT is antagonized through cytoskeleton remodeling and rearrangement of the E-cadherin/β-catenin junctions. Those changes are considered instrumental steps in promoting the reversal of the epithelial–mesenchymal transition [42], while modulation of vinculin, ROCK1, and uPA will finally antagonize the invasive/migratory proneness of breast cancer cells.

The tumor reversion we observed in our model, notwithstanding how incomplete the process can be, has the merit to highlight some essential steps that are mandatory for suppressing/achieving malignancy. Indeed, tumor reprogramming proceeds along paces that, in a reverse mode, “recapitulate” carcinogenic steps, thus allowing in ascertaining critical crossroads, which still must be investigated in depth.

The reversion process leads, sometimes, to a complete reversal but, frequently, reversion is only partially obtained. Indeed, the multistep nature of tumorigenesis is paralleled by the series of “uphill” steps required in order to achieve full reprogramming to pluripotency, and the requirement for different factors allows overcoming several barriers that are biologically designed to protect cells from the transformation, that is, to prevent cells from changing their identity. Similarly, during the reprogramming, several steps can be attained before a fully reprogrammed state could be achieved [11]. Moreover, depending on the internal/external interplay of constraints, a single molecular factor may eventually play opposite roles, as evidenced by the paradoxical behavior of the so-called oncogenes, recognized to act as either tumor promoters or tumor-suppressor depending on the permissive influence put forth by the context [43]. It is worth noting that embryo extracts promote the tumor reversion by concomitantly increasing p53 levels. In somatic cell reprogramming, it is well-recognized that the elimination of the DNA damage control checkpoint greatly boosts the efficiency of the reprogramming process [44]. Indeed, the elimination of the p53–p21 pathway by different means allows many of the starting cells to successfully complete the journey to full pluripotency. However, it does so at a price, which is that of genetic instability, in such a way that most of the induced pluripotent cells obtained in this manner carry genetic aberrations of different kinds [45]. As a result, the main potential complication in manipulating reprogrammed cells in therapeutic settings is precisely tumor generation as a result of uncontrolled growth or differentiation of the newly introduced cells into the recipient patient. Instead, in our model, down-regulation of TCTP is followed by increased activation of p53. This is a guarantee that cells cannot be further destabilized and can safely travel through the reversion pathway, until they reach a new stable, non-tumorigenic phenotype. Indeed, downstream of p53 activation, cells may be committed to apoptosis or can undergo growth arrest with subsequent differentiation, thus recovering a more physiological phenotype, and avoiding the risk to which somatic cells are exposed during reprogramming.

Treatment of breast cancer cells with 5FU is followed by a dramatic increase in apoptosis at 72 h, when cancer cells were almost all killed. However, 5FU exert only minimal, if any, effect on tumor reversion, especially when invasiveness and migrating capability are considered. Instead, addition of embryo extracts to 5FU-treated cells amplify the chemotherapy-induced cytostatic effect at early times and enhances the reversion of the invasive phenotype. This additive activity could be exploited in improving clinical response to conventional drugs, as previously reported in colon cancer patients treated with chemotherapy and embryo fish extracts [17].

A major drawback of the present study is constituted by the lack of information about the identity of the “reprogramming” factors present in the pool of molecules extracted from zebrafish embryos. However, indirect evidence suggest that they could be represented by low-molecular weight components, as they are easily absorbed by oral mucosae. Studies are currently ongoing in our laboratory to ascertain the true nature of such molecular factors.

The recognition of cancer as a disease of reprogramming opens the door to therapeutic strategies directed at correcting the wrong differentiation program in an attempt to eliminate the cancerous clone from the root. Differentiation therapies are already successfully in use for some very specific cases of cancer [46]. Our findings provide support to this new approach, highlighting that, contrary to the prevailing current “dogma”, cancer cells do not always beget cancer cells, and malignant cells may differentiate in response to (complex) environmental influences.

Tumor cells can indeed be as amenable to reprogramming as the normal ones have shown to be. Hopefully, subsequent studies will disclose the possibility to change the natural fate of tumors and, either force them to differentiate and disappear, or convert them into cells susceptible to the newly developed targeted therapies.

## 4. Materials and Methods

### 4.1. Experimental Cell Model

The human hormone-sensitive breast adenocarcinoma cell line MCF-7 (ECACC Cat# 86012803), the human Caucasian breast adenocarcinoma MDA-MB-231 (ECACC Cat# 92020424) were obtained from Sigma-Aldrich (St. Louis, MO, USA). The non-tumorigenic epithelial cell line MCF-10A (ATCC CRL-10317) were obtained from LGC Standards S.r.l, MI, Italy. Cells were seeded into 25 cm^2^ flasks (Falcon, Becton Dickinson Labware, Franklin Lakes, NJ, USA). MCF-7 and MDA-MB-231 cells were grown in Dulbecco’s modified Eagle’s medium (DMEM) supplemented with 10% fetal bovine serum (FBS) and antibiotics (penicillin 100 IU/mL, streptomycin 100 µg/mL, gentamycin 200 µg/mL; all from Euroclone Ltd., Cramlington, UK), MCF-10A were grown in Dulbecco’s modified Eagle’s medium/ nutrient mixture F12 Ham (Sigma-Aldrich, Merck, Darmstadt, Germany) supplemented with 10% horse serum (Euroclone Ltd., Cramlington, UK) and EGF 500 µ/5 mL (Santa Cruz Biotechnologies, Dallas, TX, USA), Hydrocortisone (50 µM), cholera toxin (0.5 mg/mL), insulin (10 mg/mL) (all from Sigma Chemical Co) and antibiotics (penicillin 100 IU/mL, streptomycin 100 µg/mL, gentamycin 200 µg/mL; all from Euroclone Ltd., Cramlington, UK). The cells were cultured at 37 °C in an atmosphere of 5% CO_2_ in air. The medium was changed every third day. At confluence, the cells were sub-cultured after removal with 0.05% trypsin–0.01% EDTA. In MCF-7 and MDA-MB-231 0.1 mg/mL 5-Fluorouracil (5-FU; Sigma–Aldrich), 0.1 mg/mL 5-FU + 0.3µg/mL F6 and 0.3µg/mL F6 were added in DMEM supplemented with 0.1% FBS. MCF-10A cells were firstly treated with 10ng/mL TGFβ1 (PeproTech catalog#100-21) for five days and on fifth day 0.1 mg/mL 5-FU, 0.1 mg/mL 5-FU + 0.3µg/mL F6 and 0.3 µg/mL F6 were added in F12 Ham supplemented with 0.1% horse serum.

### 4.2. Zebrafish Embryo Extracts

The embryos of Zebrafish (cultured under standard conditions as previously described [47]) were kept at different stage of development. 516 cells for each stage: blastula period (F1); 80% epiboly (F2), tailbud (F3), during the Gastrula period; 10 somites-stage (F4), 18 somites-stage (F5) and 20 somites-stage (F6), corresponding to the segmentation period, according to the developmental phases of Zebrafish embryo. Partitioning of Zebrafish embryos into the different stages of development has been assessed by three independent biologists. Measurement of total protein content for each sample and stage has been carefully recorded twice with Bradford assay. Samples were standardized (by considering protein content and number of cells) and properly stored until use. Embryos were separately collected, washed in distilled water, and dissolved with a turbo-emulsifier in cold PBS for 60 s before use.

### 4.3. In Vitro Toxicology Assay Kit Sulforhodamine B Based

4 × 10^4^ cells were seeded in a 96-multiwell and stimulated with F1, F2, F3, F4, F5, F6 at concentration of 0.1, 1, 10, 0.3, 3, 30 µg/mL, respectively. After 24, 48, and 72 h the cells were fixed for 1 h at 4 °C by gently layering 1/4 volume of cold 50% (*w*/*v*) Trichloroacetic Acid (TCA Solution) on top of the growth medium, and then rinsed with water several times to remove TCA solution, serum proteins, etc. Plates were air dried and stored until use. Blank background optical density was measured in wells incubated with growth medium without cells. The 0.4% Sulforhodamine B Solution (Sigma-Aldrich Catalog Number S2902) was added in a sufficient amount to cover the culture surface area (∼50% of the culture medium volume). Cells were stained for 20–30 min and at the end of the staining period, the stain was removed, and the cells quickly rinsed with Wash Solution (1% acetic acid) until unincorporated dye was removed. The incorporated dye was then solubilized in a volume of Sulforhodamine B Assay Solubilization Solution (10 mM Tris) equal to the original volume of culture medium. Absorbance at a wavelength of 565 nm was spectrophotometrically measured.

### 4.4. Cell Migration Assay

The 2.5 × 10^4^ cells non-stimulated (ctrl) and stimulated 0.1 mg/mL 5-FU, 0.1 mg/mL 5-FU + 0.3µg/mL F6 and 0.3µg/mL F6 respectively, were placed in 500 μL DMEM + 0.1% FBS medium (DMEM F12 + 0.1% horse serum + 10ng/mL TGF-β1 in case of MCF-10A cells) in the upper side of 8-µm filters (Falcon, BD Biosciences, San Jose, CA, USA (upper chamber) and placed in wells of a 24-well plate (Falcon, BD Biosciences) (lower chamber), containing 0.8 mL of DMEM + 10% FBS medium (DMEM F12 + 10% horse serum in case of MCF-10A cells). After 24 h of incubation, the migratory cells on the lower surface of membranes were fixed, stained with Hemacolor^®^ (HX54775574, Merck, Darmstadt, Germany) and examined microscopically cellular migration was determined by counting the number of cells on membranes in at least 4–5 randomly selected fields using a Zeiss Axiovert 10 optical microscope. For each data point, four independent experiments in duplicate were performed.

### 4.5. Cell Invasion Assay

The 2.5 × 10^4^ cells non stimulated (ctrl) and 0.1 mg/mL 5-FU, 0.1 mg/mL 5-FU + 0.3 µg/mL F6 and 0.3 µg/mL F6 as single agent respectively, were placed in 500 μL DMEM + 0.1% FBS medium (DMEM F12 + 0.1% horse serum + 10 ng/mL TGF-β1 in case of MCF-10A cells) in the upper side of 8-µm filters (BD Bio-CoatTM growth factor reduced MATRIGEL^TM^ invasion chamber, BD Biosciences-Discovery Labware, Two Oak Park, Bedford, MA, USA) (upper chamber) and placed in wells of a 24-well plate (Falcon, BD Biosciences) (lower chamber), containing 0.8 mL of DMEM 10% FBS medium (DMEM F12 + 10% horse serum in case of MCF-10A cells). After 24 h of incubation, the invasive cells on the lower surface of membranes were fixed, stained with Hemacolor^®^ (HX54775574, Merck, Darmstadt, Germany) and examined microscopically. Cellular invasion was determined by counting the number of cells on membranes in at least 4–5 randomly selected fields using a Zeiss Axiovert 10 optical microscope. For each data point, four independent experiments in duplicate were performed.

### 4.6. Cell Proliferation

MCF-7 and MDA-MB-231 cells were seeded in 6-well culture plates (Falcon, Becton Dickinson Labware, Franklin Lakes, NJ, USA) at a concentration of 1 × 10^6^ cells/well in a complete medium. The following day, the cells were refed with DMEM supplemented with 0.1% FBS containing 0.1 mg/mL 5-FU, 0.1 mg/mL 5-FU + 0.3 µg/mL F6 and 0.3 µg/mL F6. The plates were incubated for 24 h at 37 °C in an atmosphere of 5% CO_2_. Then, the cells were trypsinized and centrifuged, and cell pellets were resuspended in phosphate-buffered saline (PBS). Cell count was performed by a particle count and size analyzer (Beckman Coulter Inc., Fullerton, CA, USA). Three replicate wells were used for each data point, and the experiment was performed six times.

### 4.7. Muse™ Annexin V & Dead Cell Kit

Cells were cultured at confluence into 25 cm^2^ flasks (Falcon, Becton Dickinson Labware) in a complete medium. The apoptotic assay was performed using the Muse Annexin V and Dead cell kit (Millipore Catalog No. MCH100105). Briefly, MCF-7 and MDA-MB-231 cells were stimulated with the different embryo fish factors in DMEM 0.1% FBS. On the day of the experiment they were trypsinized, centrifuged, and resuspended in DMEM, 0.1% FBS medium to have a cell suspension between 1 × 10^5^ and 1 × 10^7^ mL^−1^. 100 μL of Muse Annexin and Dead Reagent was added. Cells were incubated for 20 min in the dark, and then analyzed with the Muse TM Cell Analyzer. Each assay was performed in triplicate.

### 4.8. Western Blots

Control and stimulated cells were washed twice with ice-cold PBS and scraped in RIPA lysis buffer (Sigma Aldrich). A mix of protease inhibitors (Complete-Mini Protease Inhibitor Cocktail Tablets, Roche, Mannheim, Germany) and phosphatase inhibitors (PhosStop; Roche, Mannheim, Germany) was added just before use. Cellular extracts were then centrifuged at 8000× *g* for 10 min. The Bradford assay was used to determine protein contents. For western blot analysis, cellular extracts were separated on SDS-polyacrylamide gels and proteins were blotted onto nitrocellulose membranes (BIO-RAD, Bio-Rad Laboratories, Hercules, CA, USA). The following antibodies were analyzed: anti-vinculin (7F9): sc-73614; anti-Rock1 (H-85): sc-5560 and anti-beta-catenin sc-7963 all from Santa Cruz Biotechnology; anti-p53 (acetyl k382) ab-75754 from Abcam; anti-TPT1 (E-AB-31729) from Elabscience; anti-E-cadherin (610181) from BD Bioscience. Antigens were detected with an enhanced chemiluminescence kit (Western Bright ECL HRP Substrate, Advansta Inc., Menlo Park, CA, USA), according to the manufacturer’s instructions. 

### 4.9. Densitometry

All Western blot images were acquired and analyzed through Imaging Fluor S densitometer (Biorad-Hercules, CA, USA). Optical density (OD) of each condition was normalized versus the signal of internal control GAPDH (anti-GAPDH #2118 from Cell Signaling Technology).

### 4.10. Confocal Microscopy

To evaluate the migratory phenotype of treated or non-treated cells, we perform the wound-healing assay using special double well culture inserts (Ibidi GmbH, Martinsried, Germany). Each insert was placed in 8-well μ-slides (Ibidi GmbH, Am Klopferspitz 19, D-82152 Martinsried, Germany) and 3.5 × 10^4^ cells were placed into both wells of each insert with 70 μL of complete medium. When cells were confluent, the culture inserts were gently removed, and cells were fed with 10% FBS DMEM (CTRL), 0.1 mg/mL 5-FU, 0.1 mg/mL 5-FU + 0.3 µg/mL F6 and 0.3 µg/mL F6 for 24 h. Then, the medium was removed and the cells were fixed with 4% paraformaldehyde for 10 min at 4 °C and washed twice for 10 min with PBS. The cells were permeabilized for 30 min using PBS, 3% BSA, 0.1% Triton X-100, followed by anti-vinculin (7F9): sc-73614, or anti anti-beta-catenin sc-7963 (all from Santa Cruz Biotechnology) staining in PBS, 3% BSA at 4 °C overnight. The cells were washed with PBS and incubated for 1 h at room temperature with appropriate secondary antibody FITC conjugated (Invitrogen Molecular Probes Eugene, OR, USA). Negative controls were processed in the same conditions besides primary antibody staining. For F-actin visualization, Rhodamine Phalloidin (Invitrogen Molecular Probes Eugene, 1: 40 dilution) was used. Cells were then washed in PBS and mounted in buffered glycerol (0.1 M, pH 9.5). Finally, analysis was conducted using a Leica confocal microscope TCS SP2 (Leica Microsystems Heidelberg GmbH, Mannheim, Germany) equipped with Ar/ArKr and He/Ne lasers. Laser line were at 543 nm and 488 nm for TRITC and FITC excitation, respectively. The images were scanned under a 40× oil objective. To analyze the colocalization of actin and vinculin, optical spatial z series composed of about 8/10 optical section with a step size of 1 μm were performed. Color channels were merged and colocalization were analyzed with the Leica confocal software.

### 4.11. Urokinase-PA Zimography

To test the enzymatic activity of urokinase plasminogen activator (uPA), aliquots of conditioned media of MDA-MB-231 human breast cancer untreated control cells, 0.1 mg/mL 5-FU, 0.1 mg/mL 5-FU + 0.3 µg/mL F6, and 0.3 µg/mL F6 treated cells were separated by electrophoresis in 10% polyacrylamide slab gels in the presence of SDS (SDS–polyacrylamide gels (PAGE)) under non-reducing conditions. The uPA was then visualized by placing the Triton-X100-washed gel on a casein–agar–plasminogen underlay. The lytic zones were plasminogen dependent. Molecular weights were calculated from the position of pre-stained markers subjected to electrophoresis in parallel lines. Densitometric scanning of zymographies was performed to derive a semi-quantitative estimation of protease activities. PA gelatin zimography was performed three times.

### 4.12. Statistical Analysis

Data were expressed as mean ± standard deviation (SD). Data were statistically analyzed with the analysis of variance (ANOVA) followed by the Bonferroni post-test. Differences were considered significant at the level of *p* < 0.05. Statistical analysis was performed by using GraphPad Instat software (GraphPad Software, Inc.; San Diego, CA, USA).

## 5. Conclusions

In the present experimental study we showed that molecular factor extracted from Zebrafish embryos isolated at the 20-somite developmental stage can reverse several malignant feature of the cancerous phenotype in a model of human breast cancer. Embryo extracts reduce cell proliferation, enhance apoptosis, and dramatically inhibit both invasiveness and migrating capabilities of cancer cells. Inhibition of migrating and invasive properties is not restricted to breast cancer cells, as embryo extracts were also effective in inhibiting the migrating phenotype adopted by normal breast cells undergoing epithelial–mesenchymal transition upon TGF-β1 stimulation. In cancerous cells embryo-induced reversion entails E-cadherin/β-catenin pathway, cytoskeleton remodeling, as well as downregulation of TCTP and the concomitant increase in p53 levels. Our findings suggest that neoplastic transformation cannot be viewed as an irreversible commitment and can be “reversed”—even partially - in response to proper morphogenetic influences.

## Supplementary Materials

Supplementary materials can be found at https://www.mdpi.com/1422-0067/20/9/2151/s1.

## References

[1] Joel M., Sandberg C.J., Boulland J.L., Vik-Mo E.O., Langmoen I.A., Glover J.C. (2013). Inhibition of tumor formation and redirected differentiation of glioblastoma cells in a xenotypic embryonic environment. Dev. Dyn..

[2] Hendrix M.J., Seftor E.A., Seftor R.E., Kasemeier-Kulesa J., Kulesa P.M., Postovit L.M. (2007). Reprogramming metastatic tumour cells with embryonic microenvironments. Nat. Rev. Cancer.

[3] Mintz B., Illmensee K. (1975). Normal genetically mosaic mice produced from malignant teratocarcinoma cells. Proc. Natl. Acad. Sci. USA.

[4] Bizzarri M., Cucina A., Biava P.M., Proietti S., D’Anselmi F., Dinicola S., Pasqualato A., Lisi E. (2011). Embryonic morphogenetic field induces phenotypic reversion in cancer cells. Curr. Pharm. Biotechnol..

[5] Biava P.M., Canaider S., Facchin F., Bianconi E., Ljungberg L., Rotilio D., Burigana F., Ventura C. (2015). Stem Cell Differentiation Stage Factors from Zebrafish Embryo: A Novel Strategy to Modulate the Fate of Normal and Pathological Human (Stem) Cells. Curr. Pharm. Biotechnol..

[6] Giuffrida D., Rogers I.M., Nagy A., Calogero A.E., Brown T.J., Casper R.F. (2009). Human embryonic stem cells secrete soluble factors that inhibit cancer cell growth. Cell Prolif..

[7] Hansis C., Barreto G., Maltry N., Niehrs C. (2004). Nuclear reprogramming of human somatic cells by xenopus egg extract requires BRG1. Curr. Biol..

[8] Ferranti F., D’Anselmi F., Caruso M., Lei V., Dinicola S., Pasqualato A., Cucina A., Palombo A., Ricci G., Catizone A. (2013). TCam-2 seminoma cells exposed to egg-derived microenvironment modify their shape, adhesive pattern and migratory behaviour: A molecular and morphometric analysis. PLoS ONE.

[9] D’Anselmi F., Masiello M.G., Cucina A., Proietti S., Dinicola S., Pasqualato A., Ricci G., Dobrowolny G., Catizone A., Palombo A. (2013). Microenvironment promotes tumor cell reprogramming in human breast cancer cell lines. PLoS ONE.

[10] Allegrucci C., Rushton M.D., Dixon J.E., Sottile V., Shah M., Kumari R., Watson S., Alberio R., Johnson A.D. (2011). Epigenetic reprogramming of breast cancer cells with oocyte extracts. Mol. Cancer.

[11] Abollo-Jiménez F., Jiménez R., Cobaleda C. (2010). Physiological cellular reprogramming and cancer. Semin. Cancer Biol..

[12] Soto A.M., Maffini M.V., Sonnenschein C. (2008). Neoplasia as development gone awry: The role of endocrine disruptors. Int. J. Androl..

[13] Cucina A., Biava P., D’Anselmi F., Coluccia P., Conti F., di Clemente R., Miccheli A., Frati L., Gulino A., Bizzarri M. (2006). Zebrafish embryo proteins induce apoptosis in human colon cancer cells. Apoptosis.

[14] Astigiano S., Damonte P., Fossati S., Boni L., Barbieri O. (2005). Fate of embryonal carcinoma cells injected into postimplantation mouse embryos. Differentiation.

[15] Tabata T., Takei Y. (2004). Morphogens, their identification and regulation. Development.

[16] Krause S., Maffini M.V., Soto A.M., Sonnenschein C. (2010). The microenvironment determines the breast cancer cells’ phenotype: Organization of MCF7 cells in 3D cultures. BMC Cancer.

[17] Downing T.L., Soto J., Morez C., Houssin T., Fritz A., Yuan F., Chu J., Patel S., Schaffer D.V., Li S. (2013). Biophysical regulation of epigenetic state and cell reprogramming. Nat. Mater..

[18] D’Anselmi F., Cucina A., Biava P.M., Proietti S., Coluccia P., Frati L., Bizzarri M. (2011). Zebrafish stem cell differentiation stage factors suppress Bcl-xL release and enhance 5-Fu-mediated apoptosis in colon cancer cells. Curr. Pharm. Biotechnol..

[19] Livraghi T., Meloni F., Frosi A., Lazzaroni S., Bizzarri M., Frati L., Biava P.M. (2005). Treatment with stem cell differentiation stage factors of intermediate-advanced hepatocellular carcinoma: An open randomized clinical trial. Oncol. Res..

[20] Proietti S., Cucina A., Giuliani A., Verna R., Palombi E., Biava P.M., Pensotti A. (2018). Fish protein extract enhances clinical response to salvage chemotherapy in colon cancer patients. Org. J. Biol. Sci..

[21] Pfeiffer M.J., Siatkowski M., Paudel Y., Balbach S.T., Baeumer N., Crosetto N., Drexler H.C., Fuellen G., Boiani M. (2011). Proteomic analysis of mouse oocytes reveals 28 candidate factors of the “reprogrammome”. J. Proteome Res..

[22] Bischof A.G., Yüksel D., Mammoto T., Mammoto A., Krause S., Ingber D.E. (2013). Breast cancer normalization induced by embryonic mesenchyme is mediated by extracellular matrix biglycan. Integr. Biol..

[23] DeCosse J.J., Gossens C.L., Kuzma J.F., Unsworth B.R. (1973). Breast cancer: Induction of differentiation by embryonic tissue. Science.

[24] Kirchberger S., Sturtzel C., Pascoal S., Distel M. (2017). Quo natas, Danio? Recent Progress in Modeling Cancer in Zebrafish. Front. Oncol..

[25] Laughlin R.B., Pines D., Schmalian J., Stojković B.P., Wolynes P. (2000). The middle way. Proc. Natl. Acad. Sci. USA.

[26] Bertolaso M., Bizzarri M., Pensotti A., Giuliani A. (2019). Co-Emergence and Collapse: The Mesoscopic Approach for Conceptualizing and Investigating the Functional Integration of Organisms. Front. Phisiol..

[27] Moirangthem A., Bondhopadhyay B., Mukherjee M., Bandyopadhyay A., Mukherjee N., Konar K., Bhattacharya S., Basu A. (2016). Simultaneous knockdown of uPA and MMP9 can reduce breast cancer progression by increasing cell-cell adhesion and modulating EMT genes. Sci. Rep..

[28] Simeoni C., Dinicola S., Cucina A., Mascia C., Bizzarri M. (2018). Systems Biology Approach and Mathematical Modeling for Analyzing Phase-Space Switch During Epithelial-Mesenchymal Transition. Methods Mol. Biol..

[29] Mierke C.T., Kollmannsberger P., Zitterbart D.P., Diez G., Koch T.M., Marg S., Ziegler W.H., Goldmann W.H., Fabry B. (2010). Vinculin facilitates cell invasion into three-dimensional collagen matrices. J. Biol. Chem..

[30] Saunders R.M., Holt M.R., Jennings L., Sutton D.H., Barsukov I.L., Bobkov A., Liddington R.C., Adamson E.A., Dunn G.A., Critchley D.R. (2006). Role of vinculin in regulating focal adhesion turnover. Eur. J. Cell Biol..

[31] Breyer J., Samarin J., Rehm M., Lautscham L., Fabry B., Goppelt-Struebe M. (2012). Inhibition of Rho kinases increases directional motility of microvascular endothelial cells. Biochem. Pharmacol..

[32] Mavria G., Vercoulen Y., Yeo M., Paterson H., Karasarides M., Marais R., Bird D., Marshall C.J. (2006). ERK-MAPK signaling opposes Rho-kinase to promote endothelial cell survival and sprouting during angiogenesis. Cancer Cell.

[33] Sivasubramaniyan K., Pal R., Totey S., Bhat V.S., Totey S. (2010). Rho kinase inhibitor y27632 alters the balance between pluripotency and early differentiation events in human embryonic stem cells. Curr. Stem Cell Res. Ther..

[34] Laeno A.M., Tamashiro D.A., Alarcon V.B. (2013). Rho-Associated Kinase Activity Is Required for Proper Morphogenesis of the Inner Cell Mass in the Mouse Blastocyst. Biol. Reprod..

[35] Mills E., LaMonica K., Hong T., Pagliaruli T., Mulrooney J., Grabel L. (2005). Roles for Rho/ROCK and vinculin in parietal endoderm migration. Cell Commun. Adhes..

[36] Grille S.J., Bellacosa A., Upson J., Klein-Szanto A.J., van Roy F., Lee-Kwon W., Donowitz M., Tsichlis P.N., Larue L. (2003). The protein kinase Akt induces epithelial-mesenchymal transition and promotes enhanced motility and invasiveness of squamous cell carcinoma lines. Cancer Res..

[37] Orsulic S., Huber O., Aberle H., Arnold S., Kemler R. (1999). E-cadherin binding prevents beta-catenin nuclear localization and beta-catenin/LEF-1-mediated transactivation. J. Cell Sci..

[38] Tuynder M., Susini L., Prieur S., Besse S., Fiucci G., Amson R., Telerman A. (2002). Biological models and genes of tumor reversion: Cellular reprogramming through tpt1/TCTP and SIAH-1. Proc. Natl. Acad. Sci. USA.

[39] Rho S.B., Lee J.H., Park M.S., Byun H.J., Kang S., Seo S.S., Kim J.Y., Park S.Y. (2011). Anti-apoptotic protein TCTP controls the stability of the tumor suppressor p53. FEBS Lett..

[40] Ito A., Kawaguchi Y., Lai C.H., Kovacs J.J., Higashimoto Y., Appella E., Yao T.P. (2002). MDM2-HDAC1-mediated deacetylation of p53 is required for its degradation. EMBO J..

[41] Amson R., Pece S., Lespagnol A., Vyas R., Mazzarol G., Tosoni D., Colaluca I., Viale G., Rodrigues-Ferreira S., Wynendaele J. (2011). Reciprocal repression between P53 and TCTP. Nat. Med..

[42] Skrypek N., Goossens S., De Smedt E., Vandamme N., Berx G. (2017). Epithelial-to-Mesenchymal Transition: Epigenetic Reprogramming Driving Cellular Plasticity. Trends Genet..

[43] Johnson D.G. (2000). The paradox of E2F1: Oncogene and tumor suppressor gene. Mol. Carcinog..

[44] Kawamura T., Suzuki J., Wang Y.V., Menendez S., Morera L.B., Raya A., Wahl G.M., Izpisúa Belmonte J.C. (2009). Linking the p53 tumour suppressor pathway to somatic cell reprogramming. Nature.

[45] Krizhanovsky V., Lowe S.W. (2009). Stem cells: The promises and perils of p53. Nature.

[46] Amson R., Karp J.E., Telerman A. (2013). Lessons from tumor reversion for cancer treatment. Curr. Opin. Oncol..

[47] Biava P.M., Carluccio A. (1997). Activation of anti-oncogene p53 produced by embryonic extracts in vitro tumor cells. J. Tumor Marker Oncol..

