# Supplementary material for: Active Fraction from Embryo Fish Extracts Induces Reversion of the Malignant Invasive Phenotype in Breast Cancer through Down-Regulation of TCTP and Modulation of E-cadherin/β-catenin Pathway
Source: Int J Mol Sci. 2019 Apr 30;20(9):2151. doi: 10.3390/ijms20092151 (PMC6539734; doi:10.3390/ijms20092151)
Supplement: Supplementary file 1 [file ijms-20-02151-s001.pdf]

Supplementary Materials

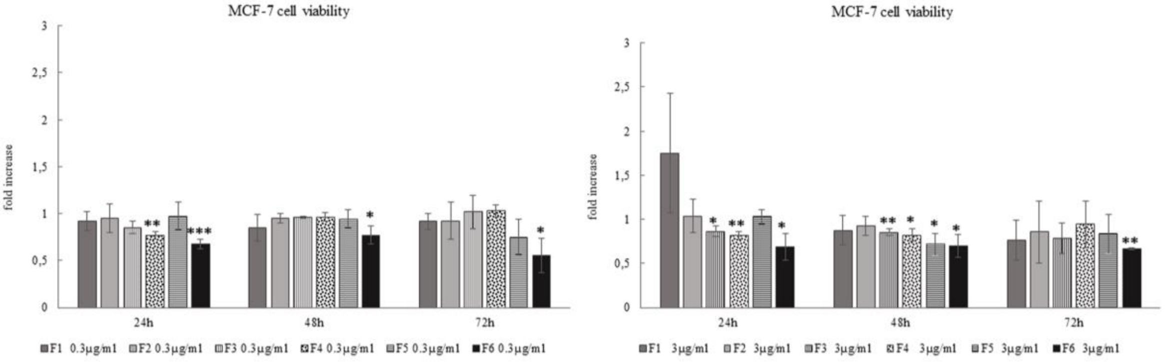

Figure S1. Sulforhodamine B (SRB) colorimetric assay performed in MCF-7 at 24 h, 48 h and 72 h. 4 × 10^4 cells were seeded in a 96-multiwell and stimulated with F1, F2, F3, F4, F5, F6 at concentration of 0.1, 1, 10, 0.3, 3, 30 µg/mL respectively. 0.4% Sulforhodamine B Solution (Sigma-Aldrich Catalog Number S2902) was added in a sufficient amount to cover the culture surface area according to the manufacturer's instructions and absorbance at a wavelength of 565 nm was spectrophotometrically measured. (mean ± S.E., $n = 3$); * $p < 0.05$, ** $p < 0.01$, *** $p < 0.001$ *vs* ctrl by the analysis of variance (ANOVA) followed by the Bonferroni post-test. Values are shown as fold increase vs ctrl.

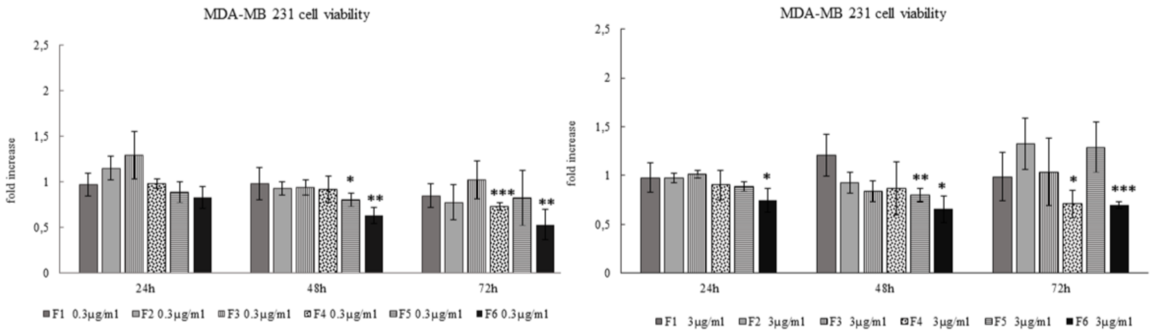

Figure S2. Sulforhodamine B (SRB) colorimetric assay performed in MDA-MB-231 at 24h, 48h and 72h. 4 × 10^4 cells were seeded in a 96-multiwell and stimulated with F1, F2, F3, F4, F5, F6 at concentration of 0.1, 1, 10, 0.3, 3, 30 µg/mL respectively. 0.4% Sulforhodamine B Solution (Sigma-Aldrich Catalog Number S2902) was added in a sufficient amount to cover the culture surface area according to the manufacturer's instructions and absorbance at a wavelength of 565 nm was spectrophotometrically measured. (mean ± S.E., $n = 3$); * $p < 0.05$, ** $p < 0.01$, *** $p < 0.001$ *vs* ctrl by the analysis of variance (ANOVA) followed by the Bonferroni post-test. Values are shown as fold increase vs ctrl.

Fig. S3

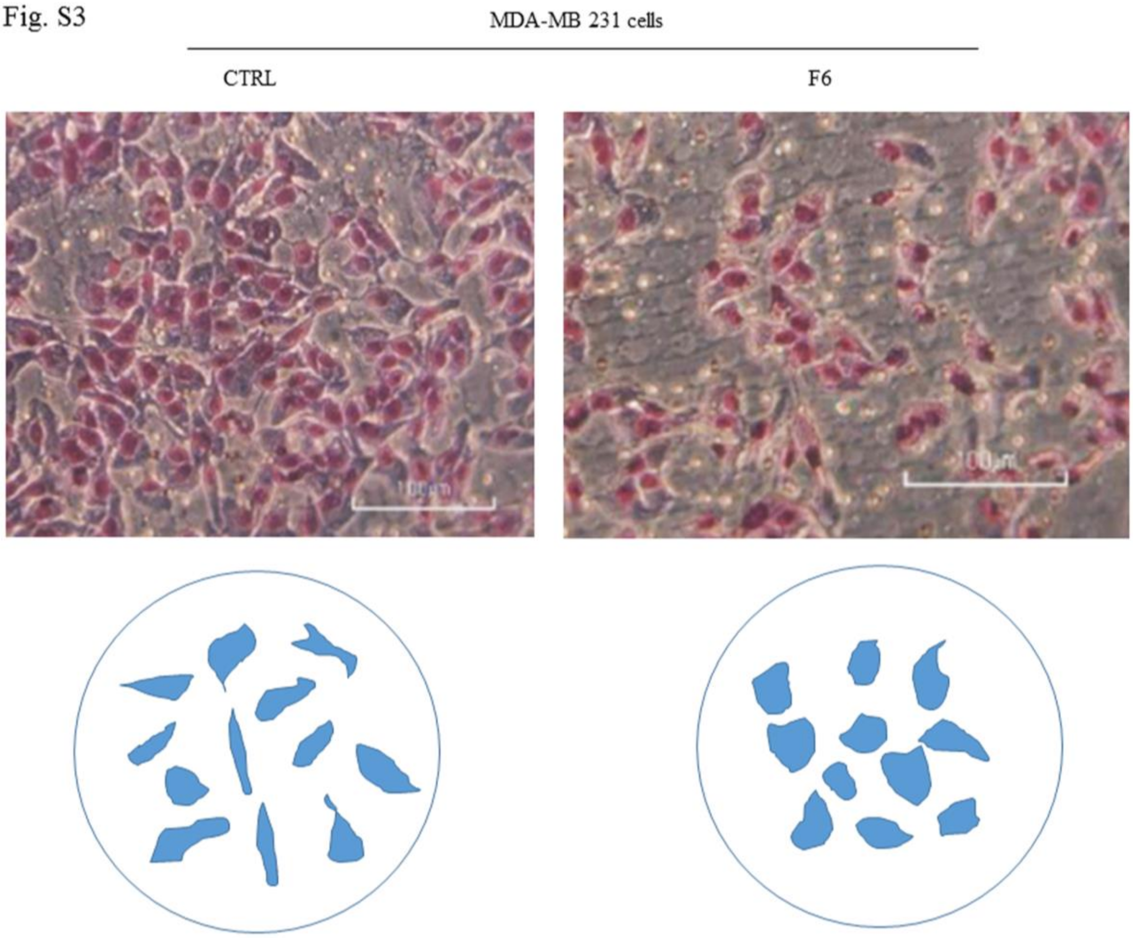

Figure 3. Embryo extracts induce cell shape remodeling. F6-treated cells recover a rounded shape, with reduced spreading and smaller nucleus in respect to control cells, characterized by elongated cytoplasm and higher nucleus/cytosol ratio. Some examples were cropped and highlighted.

Table S1. Sulforhodamine B (SRB) colorimetric assay performed in MCF-7 at 24h and 72h.

	**MCF-7 viability test 24h**					
	**0.1 µg/ml**	**1 µg/ml**	**10 µg/ml**	**0.3 µg/ml**	**3 µg/ml**	**30 µg/ml**
**ctrl**	1 ± 0.01	1 ± 0.01	1 ± 0.01	1±0.02	1±0.02	1±0.02
**F1**	1.05±0.02	1.05±0.12	1.19±0.02	0.85±0.1*	0.79±0.07**	0.77±0.05**
**F2**	1.02±0.31	1.23±0.07	1.14±0.06	0.85±0.06**	0.85±0.07*	0.81±0.01***
**F3**	1.08±0.16	1.17±0.11	1.03±0.12	0.81±0.03***	0.81±0.04***	0.82±0.09*
**F4**	0.93±0.05*	0.95±0.05	0.80±0.12*	0.80±0.12**	0.85±0.03***	0.78±0.02***
**F5**	1.22±0.36	1.20±0.24	1.29±0.17	1.08±0.1	0.97±0.07	1.03±0.1
**F6**	0.86±0.07**	1.13±0.34	1.25±0.07	0.72±0.1**	0.07±0.05***	0.78±0.17

	**MCF-7 viability test 72h**					
	**0.1 µg/ml**	**1 µg/ml**	**10 µg/ml**	**0.3 µg/ml**	**3 µg/ml**	**30 µg/ml**
**ctrl**	1.0±0.03	1.0±0.03	1.0±0.03	1.0±0.01	1.0±0.01	1.0±0.01
**F1**	1.04±0.11	0.96±0.27	0.97±0.11	1.17±0.22	1.28±0.17	0.84±0.04***
**F2**	1.30±0.12	0.93±0.13	1.13±0.12	1.05±0.03	1.25±0.11	0.79±0.12*
**F3**	1.38±0.44	1.08±0.20	1.04±0.29	1.13±0.12	1.10±0.18	0.97±0.12
**F4**	1.07±0.27	1.05±0.24	0.76±0.13*	1.06±0.07	1.07±0.17	0.83±0.05**
**F5**	0.85±0.47	0.79±0.26	1.0±0.52	0.54±0.12**	0.80±0.27	1.13±0.34
**F6**	0.60±0.08**	0.61±0.37	0.56±0.05***	0.34±0.15**	0.68±0.13**	0.59±0.06***

4×10^4 cells were seeded in a 96-multiwell and stimulated with F1, F2, F3, F4, F5, F6 at concentration of 0.1, 1, 10, 0.3, 3, 30 µg/mL respectively. 0.4% Sulforhodamine B Solution (Sigma-Aldrich Catalog Number S2902) was added in a sufficient amount to cover the culture surface area according to the manufacturer's instructions and absorbance at a wavelength of 565 nm was spectrophotometrically measured. (mean ± S.E., $n = 3$); * $p < 0.05$, ** $p < 0.01$, *** $p < 0.001$ *vs* ctrl by the analysis of variance (ANOVA) followed by the Bonferroni post-test. Values are shown as fold increase vs controls (ctrl).

Table S2. Sulforhodamine B (SRB) colorimetric assay performed in MDA-MB-231 at 24 h and 72 h.

	MDA-MB-231 viability test 24h					
	0.1 µg/ml	1 µg/ml	10 µg/ml	0.3 µg/ml	3 µg/ml	30 µg/ml
ctrl	1 ± 0.01	1 ± 0.01	1 ± 0.01	1±0.001	1±0.001	1±0.001
F1	1.12±0.15	0.95±0.05	1.05±0.1	0.90±0.20	0.90±0.16	1.33±0.38
F2	1.15±0.001	0.98±0.09	0.99±0.04	1.24±0.05	1.06±0.21	0.88±0.10*
F3	1.12±0.05	1.08±0.23	0.90±0.11	1.11±0.05	1.18±0.25	1.01±0.13
F4	0.79±0.27	1.17±0.28	0.88±0.07*	1.02±0.09	0.99±0.05	0.99±0.20
F5	0.72±0.17*	1.15±0.33	0.97±0.29	0.97±0.07	0.91±0.09	1.04±0.37
F6	0.84±0.05**	0.72±0.19	0.86±0.28	0.70±0.09**	0.79±0.24	0.94±0.24

	MDA-MB-231 viability test 72h					
	0.1 µg/ml	1 µg/ml	10 µg/ml	0.3 µg/ml	3 µg/ml	30 µg/ml
ctrl	1.0±0.001	1.0±0.001	1.0±0.001	1.0±0.01	1.0±0.01	1.0±0.01
F1	1.63±0.13	1.24±0.23	1.56±0.06	0.92±0.08***	0.79±0.08**	2.18±0.83
F2	1.40±0.50	1.30±0.13	1.34±0.18	0.56±0.08	0.91±0.13	1.86±0.63
F3	0.80±0.26	0.78±0.14*	0.87±0.15	0.86±0.09*	0.88±0.18	1.65±0.38
F4	0.79±0.28	0.56±0.03***	0.60±0.10**	0.69±0.18*	0.58±0.11**	1.37±0.36
F5	0.56±0.22*	0.76±0.15	0.84±0.23	0.68±0.14*	1.08±0.66	1.71±0.26
F6	0.46±0.13**	0.78±0.25	0.58±0.33	0.44±0.10***	0.72±0.21**	1.92±0.43

4×10^4 cells were seeded in a 96-multiwell and stimulated with F1, F2, F3, F4, F5, F6 at concentration of 0.1, 1, 10, 0.3, 3, 30 µg/mL respectively. 0.4% Sulforhodamine B Solution (Sigma-Aldrich Catalog Number S2902) was added in a sufficient amount to cover the culture surface area according to the manufacturer's instructions and absorbance at a wavelength of 565 nm was spectrophotometrically measured. (mean ± S.E., $n = 3$); * $p < 0.05$, ** $p < 0.01$, *** $p < 0.001$ *vs* ctrl by the analysis of variance (ANOVA) followed by the Bonferroni post-test. Values are shown as fold increase vs ctrl.
